# Non-coding RNAs as Putative Biomarkers of Cancer-Associated Cachexia

**DOI:** 10.3389/fcell.2020.00257

**Published:** 2020-04-21

**Authors:** Sara Donzelli, Alessia Farneti, Laura Marucci, Federica Ganci, Andrea Sacconi, Sabrina Strano, Giuseppe Sanguineti, Giovanni Blandino

**Affiliations:** ^1^Oncogenomic and Epigenetic Unit, IRCCS Regina Elena National Cancer Institute, Rome, Italy; ^2^Radiotherapy Unit, IRCCS Regina Elena National Cancer Institute, Rome, Italy; ^3^UOSD Clinical Trial Center, Biostatistics and Bioinformatics, IRCCS Regina Elena National Cancer Institute, Rome, Italy; ^4^SAFU Unit, IRCCS Regina Elena National Cancer Institute, Rome, Italy

**Keywords:** miRNA, cachexia, liquid biopsy, HNSCC (head and neck squamous cell carcinoma), myomiR

## Abstract

Cachexia is a complex metabolic syndrome that determines a severe body weight loss characterized by a marked reduction in muscle mass. About 80% of patients with advanced cancer develop cachexia due to both the tumor itself and cancer treatment (radiotherapy and/or chemotherapy), which is associated to a worse prognosis. Despite its clinical relevance, this syndrome is still under-diagnosed and it lacks effective treatments. Radio-chemotherapy treatment is essential in patients with advanced head and neck cancers (HNSCC). Although this treatment has improved patients’ life expectancy, it has also dramatically increased their need for assistance and support. The management of adverse symptoms, including cachexia, is of great importance in order to avoid delays in therapy, reduction of dosages and hospitalizations. MicroRNAs (miRNAs) are small non-coding RNA molecules, which have emerged as powerful biomarkers in stratifying human cancers. Due to their high stability in body fluids, miRNAs might be excellent non-invasive biomarkers for the early detection and follow-up of cancer patients. Here, we will summarize the current knowledge and debate the strong need to identify circulating biomarkers for the early diagnosis of cachexia. We will propose circulating non-coding RNAs as biomarkers for detecting early cachexia and implementing specific treatment. We will also discuss the potential use of circulating miRNAs as biomarkers of cachexia in HNSCC patients’ blood samples collected before and after radio-chemotherapy treatment. Our intent is to pave the way to the identification of specific circulating miRNAs associated to cachexia occurrence and to the design of specific interventions aimed at improving the quality of life of cancer patients.

## Cancer-Associated Cachexia in Advanced Cancer Patients

Cachexia is a complex multifactorial syndrome characterized by the loss of mass and functionality of skeletal muscle and by the loss of adipose tissue, with consequent progressive loss of body weight (DeWys, 1982). Unlike malnutrition, cachexia cannot be resolved simply with conventional nutritional support (Fearon et al., 2011).

This syndrome affects the majority of patients with advanced cancer treated with radio-chemotherapy, including in particular patients with advanced head and neck cancers (HNSCC) (Gorenc et al., 2015; Kwon et al., 2017). It is also associated with a poor prognosis, an altered quality of life and a reduced tolerance and response to anticancer therapies (DeWys, 1982; Fearon et al., 2011).

Weight loss is a common feature in patients with HNSCC and can occur before, during and after radio-chemotherapy treatment.

The correct diagnosis of cachexia and the maintenance of muscle mass represent an important goal in the care of cancer patients.

The development of cachexia varies according to the nature, the stage and the site of the tumor, the type of treatment, but it is also based on individual characteristics (genetic predisposition, initial BMI and body composition, physical activity, food intake, comorbidity and gut microbiota) (Bindels and Delzenne, 2013; Johns et al., 2013). Therefore, due to the heterogeneity of its manifestation and to the lack of a precise definition, the diagnosis of cachexia is often not performed promptly.

The need for an early diagnosis determines the need to identify biomarkers able to reflect the process of muscular atrophy that characterizes cachexia. The ideal biomarker should be easy to quantify without the need for an invasive muscle biopsy.

## Current Knowledge About Potential Biomarkers for Cancer-Associated Cachexia

Cancer-associated cachexia is a very complex and still poorly characterized syndrome whose molecular pathways remain to be still elucidated.

Muscle atrophy, that characterizes cachexia, is the result of an imbalance between muscle protein synthesis and degradation that determine a decrease in myofibrillar and sarcoplasmic proteins leading to muscle fibers shrinkage.

What is taken for granted is the key role of systemic inflammation in the occurrence of cachexia, determined both by the presence of the tumor itself and by the host-derived factors (Argiles et al., 2019).

For this reason, to date, the majority of the studies aimed at the discovery of powerful biomarkers for cachexia focused on pro-inflammatory cytokines released by tumor, immune and stromal cells.

In particular the cytokine tumor necrosis factor α (TNFα), previously called cachectin, has been demonstrated to be a major player in cancer related cachexia as it is able to induce muscle wasting through NFkB pathway (Han et al., 1999).

Different studies in animal models and cancer patients demonstrated that high levels of interleukin 6 (IL-6) correlate with muscle wasting, inhibition of protein synthesis, promotion of protein degradation and autophagy in myotubes (White et al., 2011; Pettersen et al., 2017).

Supporting the key role of inflammation in cancer-related cachexia occurrence, recently Penafuerte et al. (2016) reported an increase in neutrophil-derived proteases (NDPs), angiotensin II (Ang II), transforming growth factor beta 1 (TGFβ1) and C-reactive protein (CRP) plasma levels in cachectic and pre-cachectic cancer patients.

Collectively, the release of these pro-inflammatory cytokines has been demonstrated to promote ubiquitin-proteasome and autophagy lysosome pathways in skeletal muscle cells thereby determining muscle wasting.

Despite these evidences, there is still an incomplete understanding of the underlying biology of cancer-associated cachexia, and there is still the need to find powerful biomarkers useful for the diagnosis and management of such complex metabolic syndrome.

## MicroRNAs

MicroRNAs (miRNAs) are highly conserved single strand RNA molecule of about 17–22 nucleotides in length whose role in the regulation of wide range of biological processes has been widely characterized (Gebert and MacRae, 2019).

**FIGURE 1:**
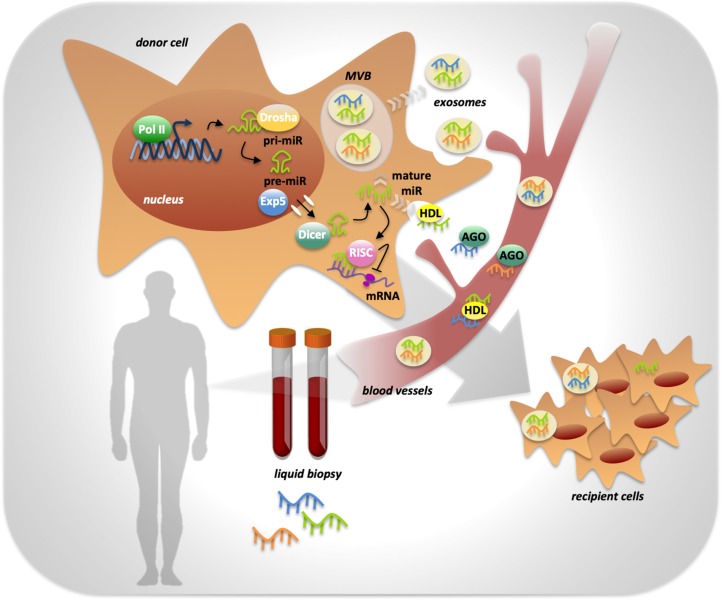
miRNAs processing, activity and release in body fluids. MiRNA genes are transcribed by RNA polymerase II into a primary transcript with a hairpin double helix structure of about 300 nucleotides called pri-miR. The endonuclease Drosha is responsible of pri-miR processing into a smaller precursor called pre-miR, that is exported from the nucleus to the cytoplasm by Exportin 5 enzyme (Exp5). Into the cytoplasm the endonuclease Dicer completes miRNA biogenesis by processing the pre-miR in the mature miRNA. miRNA activity, consisting in the inhibition of target mRNAs translation, is determined by its inclusion in the RISC complex. Mature miRNAs can be released by the cell into the extracellular space in two ways: through the inclusion in protein complexes or in exosomes that preserve them from RNases activity. Once in body fluids, miRNAs can reach distant sites in the body and act as mediators of cell-to-cell communication. miRNAs’ peculiar high stability makes them ideal powerful biomarkers easily detectable trough liquid biopsy.

miRNAs’ biogenesis starts with the transcription by RNA polymerase II that generates a primary transcript with a hairpin double helix structure of about 300 nucleotides called pri-miR (Treiber et al., 2019). The pri-miR is processed by the endonuclease Drosha and the cofactor DGCR8 into a smaller precursor called pre-miR in the nucleus. The pre-miR is exported to the cytosol by the exportin 5 enzyme (Treiber et al., 2019). In the cytosol, the endonuclease Dicer cleaves the pre-miR to generate the miRNA duplex, of which only one strand will be the mature miRNA (Treiber et al., 2019). The mature miR is then included in the RNA-induced silencing complex (RISC), through which it is brought to the target mRNAs (Treiber et al., 2019) (Figure 1).

The peculiar mechanism of action of miRNAs consists in the ability to bind to the 3′ UTR of their target mRNAs and to promote their degradation or the inhibition of mRNA translation. This leads to a decrease of the expression of specific proteins.

Due to the multiplicity of mRNAs target for each miRNA, fine modulation in their expression levels can determine relevant changes in the cell. Indeed miRNAs’ deregulation may be the cause of several pathological conditions, such as cancer (Tufekci et al., 2014).

## MicroRNAs as Potential Cancer Biomarkers in Tissues and in Body Fluids

Growing evidence has demonstrated the broad implication of miRNAs in cancer occurrence and allowed the classification of miRNAs in two main categories according to their target mRNAs: the so called “oncomiRNAs” and the “tumor suppressor miRNAs” (Fearon et al., 2011; Blandino et al., 2014; Frixa et al., 2015; Verduci et al., 2019). Under physiological conditions there is a balance between these two classes of miRNAs. At the onset of cancer there are alterations that can unbalance the ratio between oncomiRNAs and tumor suppressor miRNAs fostering tumorigenesis. Deregulation of miRNAs expression can be established by alterations that affect their biogenesis, such as: (a) epigenetic modification on their regulatory regions; (b) changes in the activity of specific transcription factors; (c) alterations in processing enzymes involved in miRNAs’ maturation steps (mainly Drosha and Dicer) (Rakheja et al., 2014; Lin and Gregory, 2015; Frixa et al., 2018; Ramassone et al., 2018).

The initial studies, concerning the role of miRNAs in cancer occurrence, focused on patients-derived tumoral tissues and allowed the identification of specific signature able to stratify human cancers (Lu et al., 2005; Calin and Croce, 2006; Di Leva and Croce, 2013). In particular, miRNAs discriminate tumoral from non-tumoral tissues, or metastatic from non-metastatic tumors, or different histotypes of the same tumor type, or, in treated patients, responder from non-responder individuals (Donzelli et al., 2015; Lindholm et al., 2019; Sokilde et al., 2019).

One of the most characterized oncogenic miRNAs is miR-21, whose expression has been found to be up-regulated in several solid and hematological malignancies (Feng and Tsao, 2016). The widespread pro-tumorigenic function of miR-21 depends on its ability to bind to the 3′UTR of several tumor suppressor mRNAs independently from the tissue context. Its oncogenic activities include promotion of cell proliferation, migration, invasion, metastasis and resistance to chemotherapeutic treatments (Zhu et al., 2008; Pfeffer et al., 2015). Moreover, miR-21 has been demonstrated to be an important biomarker of poor prognosis in several cancer types (Yan et al., 2008; Zhou et al., 2014; Arantes et al., 2017; Labib et al., 2017). For this reasons miR-21 represents one of the actionable miRNAs for novel therapeutic strategies and it is considered a robust biomarker to be implemented in clinical practice (Bonneau et al., 2019; Hanna et al., 2019).

Conversely, miR-145-5p is one of the most studied tumor-suppressor miRNAs as it results to be down-regulated in several types of cancer (Cui et al., 2014; Donzelli et al., 2015). Most of the findings revealed that miR-145-5p regulates the expression of several oncogenes in particular genes involved in cell invasion, migration and metastatization (Ye et al., 2019). Notably, the attempt to disclose a therapy for the replacement of miR-145-5p in tumor cells is actively pursued. These and other studies highlighted the promising role of miRNAs as powerful biomarkers for cancer diagnosis and prognosis and as novel actionable targets for more effective cancer therapies.

Since 2008, with the first evidence of the presence of miRNAs in plasma and in others biological fluids, and thus in the extracellular environment, the potential role of miRNAs as mediators of cell-to-cell communication has been deeply investigated (Mitchell et al., 2008; Kosaka et al., 2010; Li et al., 2019).

Indeed, it has been demonstrated that miRNAs exert paracrine functions and are essential for tissue communication.

In regard of cancer, the release of miRNAs by tumoral cells in the extracellular environment has been assessed for several types of cancer (Izzotti et al., 2016).

The release of miRNAs from tumoral cells can occur in two different ways: miRNAs can be included in microvesicles that are released from the cells through blebbing of the plasma membrane, otherwise cells can actively release miRNAs in microparticle-free form and they can bind to high-density lipoproteins or to RNA-binding proteins such as Ago2 (Kosaka et al., 2013) (Figure 1).

A peculiar feature of circulating miRNAs is their remarkable stability, due to their small size and to the inclusion in protein complexes or in microvesicles that preserve them from RNase activity. Indeed, miRNAs are present in almost all biological fluids such as plasma, serum, saliva, urine, cerebrospinal liquid, milk, amniotic fluid and tear (Cortez et al., 2011; Izzotti et al., 2016).

MiRNAs’ high stability, the non-invasive way to collect (i.e., blood collection), to detect and to quantify (i.e., Real-Time PCR) them, are all features that overlap to those of an ideal biomarker. Indeed, the majority of the studies, aimed to the discovery of novel powerful biomarkers for cancer screening, diagnosis, prognosis and monitoring of the effectiveness of therapies in cancer patients, are focusing on genome wide approaches for the evaluation of miRNAs in body fluids (Hamam et al., 2017; Cui et al., 2019).

Intriguingly, miR-21 represents one of the circulating miRNAs whose concentration is increased in the serum of patients with the most varied types of cancer, suggesting the potential use of miR-21 as non-invasive diagnostic markers (Wu et al., 2015; Pulito et al., 2017; Cui et al., 2019).

## Are Skeletal Muscle MicroRNAs Potential Biomarkers for Cancer-Associated Cachexia?

Several genome wide studies revealed that miRNAs are differentially expressed in human tissues. In particular, a small group of miRNAs, referred to as *myomiRs*, that are enriched or exclusively expressed in the striated muscle has been identified. This muscle-specific group of miRNAs includes miR-1, miR-133a, miR-133b, miR-206, miR-208a, miR-208b, miR-486, and miR-499 (Kirby and McCarthy, 2013). The tissue-specificity is due to the presence of muscle-specific transcription factor binding sites on the regulatory regions of these miRNAs and, for some of them, to the genomic localization within the myosin heavy chain (MyHC) genes. The role of myomiRs in the regulation of muscle homeostasis, development and functionality has been extensively characterized (Horak et al., 2016; Sjögren et al., 2017). Moreover, deregulation of their expression resulted to be associated with muscle atrophy, one of the main hallmark of cancer-associated cachexia (Suzuki and Springer, 2018).

This suggests the possibility for myomiR to be novel mediators and powerful biomarkers for cancer-associated cachexia. In the last years, different studies on muscle tissue from mice models of cachexia or from cachectic cancer patients revealed a correlation between myomiRs deregulation and cachexia occurrence and maintenance (Soares et al., 2014; Lee et al., 2017; Fernandez et al., 2019).

The muscle-specific miR-206, together with miR-21, was one of the first miRNAs identified to be positively associated to muscle wasting by performing miRNA profiling of muscles derived from different atrophic mice models (Soares et al., 2014).

Recently, Lee et al. (2017), by performing miRNA sequencing from tibialis anterior muscles of cachectic lung carcinoma mice and of healthy mice, identified a signature of 9 miRNAs differentially expressed: miR-147, miR-205-3p, miR-229a3p, miR-233-3p, miR-431-5p, miR-511-3p, miR-665-3p, miR-1933-3p, and miR-3473d. Gene ontology analysis of these miRNAs indicated an involvement in cellular development, cell cycle, cell morphology, cell death and survival, and inflammatory responses, supporting their role in muscle wasting (Lee et al., 2017).

More recently, Fernandez and collaborators, performed an integrated genome wide analysis by combining miRNA/mRNA sequencing from the same set of skeletal muscles derived from mice models of cancer-associated cachexia (Fernandez et al., 2019). In particular, they identified 18 miRNAs differentially expressed in skeletal muscle of cachectic mice compared to the controls. Among the 18 miRNAs, 13 resulted to be up-regulated and 5 to be down-regulated. The integrative analysis allowed the authors to generate a miRNA-mRNA network composed of 171 interactions between 18 miRNAs and 131 target mRNAs. This analysis revealed enrichment for genes involved in extracellular matrix organization, highlighting their contribution to cancer-associated cachexia (Fernandez et al., 2019).

Genome wide studies performed in muscles derived from cachectic cancer patients enlarged the knowledge about miRNAs’ contribution to cancer-associated cachexia.

Interestingly, Narasimhan et al. (2017) analyzed miRNAs expression of human skeletal muscle in cachectic and non-cachectic pancreatic and colorectal cancer patients and identified 8 novel deregulated miRNAs: miR-3184-3p, miR-423-5p, let-7d-3p, miR-1296-5p, miR-345-5p, miR-532-5p, miR-423-3p, and miR-199a-3p. These miRNAs resulted to be up-regulated in skeletal muscle of cachectic patients, and pathway analysis of their potential mRNA targets identified pathways related to myogenesis and inflammation (Narasimhan et al., 2017).

More recently, Van de Worp et al. (2019), by performing miRNA profiling in skeletal muscle of cachectic lung cancer patients compared to matched healthy controls, identified a signature of 28 miRNAs differentially expressed. Interestingly, 4 miRNAs our of 5 which resulted to be up-regulated in cachetic patients, belong to the same cluster (miR-450a-5p, miR-450b-5p, miR-424-5p, and miR-424-3) (Van de Worp et al., 2019).

Adipose tissue loss also occurs in cancer-associated cachexia. This feature has been scarcely investigated. Kulyte et al. (2014) identified a signature of 5 miRNA differentially expressed in abdominal subcutaneous adipose tissue from gastrointestinal cancer patients with or without cachexia. In particular, miR-483-5p, miR-23a, miR-744, and miR-99b were down-regulated, whereas miR-378 was significantly up-regulated in cachectic patients. In details, the authors demonstrated that miR-378 up-regulation in adipose tissue is required to promote catecholamine-stimulated lipolysis.

## Potential Use of Circulating MicroRNAs as Biomarkers for Cancer-Associated Cachexia

Despite genome wide studies performed either in muscle or adipose tissues highlight the involvement of miRNAs in cancer-associated cachexia, the validation in large cancer patient cohorts is lacking and the molecular mechanisms underlying their specific involvement need to be deeply elucidated (Table 1).

**TABLE 1:** miRNAs demonstrated to be related to cancer-associated cachexia.

Tissue miRNAs	Tissue	References
miR-206, miR-21	Mice muscles	Soares et al., 2014
miR-147, miR-205-3p, miR-229a3p, miR-233 3p, miR-431-5p, miR-511-3p, miR-665-3p, miR-1933-3p, miR-3473d	Mice muscles	Lee et al., 2017
miR-10b-5p, 1249-3p, miR-144-3p, miR-144-5p, miR-146a-5p, miR-146b-5p, miR-181c-3p, miR-183-5p, miR-1843a-3p, miR-223-3p, miR-29b-3p, miR-338-5p, miR-350-3p, miR-3535, miR-379-3p, miR-382-5p, miR-451a, miR-671-3p	Mice muscles	Fernandez et al., 2019
miR-3184-3p, miR-423-5p, let-7d-3p, miR-1296-5p, miR-345-5p, miR-532-5p, miR-423-3p, miR-199a-3p	Human skeletal muscle	Narasimhan et al., 2017
miR-483-5p, miR-23a, miR-744 and miR-99b, miR-378	Human skeletal muscle	Kulyte et al., 2014
miR-450a-5p, miR-424-5p, miR-450b-5p, miR-424-3p, miR-335-3p, miR-103-3p, miR-483-5p, mir-409-3p, miR-15b-5p, miR-370-3p, miR-20a-3p, miR-451a, miR-517c-3p, miR-144-5p, miR-766-3p, miR-1255b, miR-517a-3p, miR-512-3p, miR-522-3p, miR-520g-3p, miR-483-3p, miR-519a-3p, miR-26a-2-3p, miR-485-3p, miR-379-5p, miR-518b, miR-520h, miR-656-3p	Human skeletal muscle	Van de Worp et al., 2019

**Circulating miRNAs**	**Type of tumor**	**References**

miR-1	Advanced hepatocellular carcinoma	Koberle et al., 2013
miR-486	Breast cancer	Chen et al., 2014
miR-21	Colorectal cancer	Okugawa et al., 2018
miR-203	Colorectal cancer	Okugawa et al., 2019
miR-130a	Head and neck cancer patients	Powrozek et al., 2018

Moreover, reasoning about muscle or adipose tissue miRNAs as potential biomarkers for cancer-associated cachexia is poorly feasible, because biopsies are too invasive for routine analysis and cannot be applied for the monitoring of cachexia during cancer patients treatment. Therefore, there is the urgent need to identify alternative strategies and design clinical studies to validate miRNAs as potential biomarkers of cancer associated cachexia. Recent studied have started to look at body fluid circulating miRNAs as promising and powerful biomarkers for the diagnosis and the monitoring of cancer-associated cachexia (Table 1).

In particular, several studies have reported that low concentrations of myomiRs in blood correlate with poor prognosis in non-small cell lung cancer, melanoma, astrocytoma, or osteosarcoma patients (Hu et al., 2010; Li et al., 2014; Zhang C. et al., 2014; Tian et al., 2015). This because skeletal muscle, under physiological conditions, continuously releases exosomes contained myomiRs in the blood (Guescini et al., 2015), but in advanced cancers, when a substantial reduction of muscle mass occurs, there is a consequent decrease in circulating myomiRs. Indeed, Koberle et al. (2013) demonstrated that low levels of circulating miR-1 associated with cachexia in advanced hepatocellular carcinoma patients. Moreover, Chen et al. (2014) reported the circulating muscle enriched miR-486 to be lower in breast cancer patients compared with healthy subjects. More recently, Van Westering et al. (2019), demonstrated that the amount of released myomiR in the serum is dependent on dystrophin protein levels and on its distribution at the sarcolemma.

Not only myomiRs, but also miRNAs released by the tumor itself hold the promise to be considered in the next future robust circulating biomarkers for cancer-associated cachexia. Originally, Fabbri et al. (2012) demonstrated that miR-21 and miR-29a released by lung cancer cells bind to receptors of the Toll-like receptor (TLR) family (murine TLR7 and human TLR8) in immune cells; thereby promoting a TLR-mediated pro-metastatic inflammatory response. As inflammation is one of the main causes of muscle wasting in cancer-associated cachexia, circulating miR-21 and miR-29a seem to be good candidates for cachexia diagnosis and monitoring. The potential use of circulating miR-21 as biomarker for cancer-associated cachexia is supported by other two studies (He et al., 2014; Okugawa et al., 2018). In particular, He et al. (2014) reported that miR-21 contained in lung cancer- and pancreatic cancer-derived microvesicles was able to induce TLR7-mediated cell death in murine myoblasts. More recently, Okugawa et al., demonstrated that circulating miR-21 levels increased in cachectic colorectal cancer patients (Okugawa et al., 2018). Recently they also reported that circulating miR-203 is able to predict myopenia in metastatic colorectal cancer patients (Okugawa et al., 2019).

Looking at the circulating miRNAs that can predict cachexia in radiotherapy-treated head and neck cancer patients, Powrozek et al. (2018), recently identified miR-130a as a good candidate. Indeed, by evaluating the circulating levels of miR-130a in 70 head and neck cancer patients, they observed that patients with low levels of miR-130a were at higher risk to be classified as cachectic, compared to those with high levels of miR-130a. In agreement with a previous study that reported the cytokine TNFα to be a direct target of miR-130a in cervical cancer cell lines (Zhang J. et al., 2014), the authors observed a correlation between low levels of miR-130a and high plasma levels of TNFα in head and neck cancer patients, confirming the key role of inflammation in the onset of cancer-associated cachexia.

The hypothesis that circulating miRNAs could represent powerful biomarkers for the diagnosis and monitoring of cancer-associated cachexia appears to have a strong rationale. In particular, due to the relevant contribution of cancer treatments to cachexia, we aim to evaluate the role of circulating miRNAs in a cohort of advanced head and neck squamous cell carcinoma (HNSCC) patients treated with radio-chemotherapy. To this purpose we firstly performed a retrospective analysis on sera samples of 15 patients enrolled in a prospective study funded by Italian Association for Cancer Research (AIRC, project No.17028) with pathologically confirmed squamous cell carcinoma of the oropharynx, Karnofsky Performance Status >80, stage III or IV without distant metastases, treated with radiotherapy + chemotherapy. Cigarette smoking, alcohol consumption and p16/HPV status, basal weight and variation during treatment, were recorded.

**FIGURE 2:**
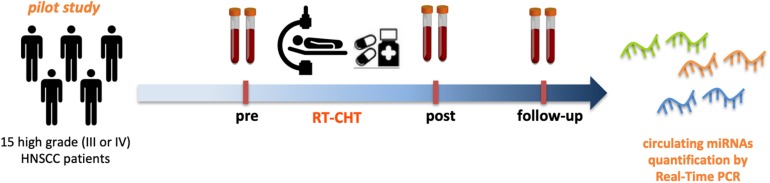
Circulating miRNAs as powerful biomarkers for radio-chemotherapy treatment related cachexia in HNSCC patients. Pilot study design. Serum samples from 15 high grade (III or IV) HNSCC patients before (pre) and after (post- and follow-up) radio-chemotherapy treatment have been collected and subjected to miRNAs expression analysis by Real-Time PCR to asses the feasibility in the quantification of circulating miRNAs.

Patient sera samples (*n* = 15) were collected before the treatment (pre) and 3 months after the treatment (post). Sera collected at 6 months after the treatment (follow-up) were available for 8 out of 15 patients (Figure 2).

As a proof of concept we evaluated, by Real-Time PCR, the expression levels of miR-21, due to the previously mentioned evidence about its potential use as biomarker for cancer-associated cachexia. This analysis allowed us to test the quality of the samples and to confirm the presence of miRNAs in the serum of patients affected by head and neck tumors. In particular we confirmed that miR-21 is highly expressed in sera samples of HNSCC patients and it is modulated by radio-chemotherapy treatment.

Prospectively we aim to evaluate the expression of circulating miRNAs (in particular miR-21 and myomiRs) in a prospective cohort of patients with histologically confirmed squamous cell carcinoma of the oral cavity, oropharynx, hypopharynx and larynx, stage III o IV, treated with definitive or adjuvant radio-chemotherapy. The parameters related to cachexia will be evaluated (i.e., weight and its variation over time, variation Body Mass Index, determination of the degree of systemic inflammation by measuring proinflammatory cytokines). Furthermore, the assessment of nutritional status, fatigue, quality of life through questionnaires, will be recorded at baseline during and at follow-up, in order to identify predictive biomarkers of the onset and progression of cachexia.

## Conclusion

Cancer-associated cachexia is a serious limitation for cancer patients that severely worsen their quality of life, especially for those treated with radio-chemotherapy. Up to date there are no effective treatments for this complex metabolic syndrome. This is mainly due to the lack of a deep characterization of the molecular mechanisms underlying cachexia.

The unmet clinical need that makes very difficult to diagnose timely and properly cachexia is the lack of specific biomarkers. Inflammatory cytokines, such as TNFα and IL-6 might be envisaged as biomarkers for diagnosis and monitoring of cachexia, but further confirmatory studies using larger casuistries than those already analyzed are needed.

MiRNAs are emerging as promising biomarkers not only in cancer but also in other diseases due to their involvement in key biological processes and to their higher stability.

Recently, the evidence of the involvement of miRNAs in cancer-associated cachexia is emerging. Despite the findings are still preliminary they might pave the way for the consideration of miRNAs as useful tools in the diagnosis and monitoring of cachexia.

## Author Contributions

SD, SS, and GB contributed to the writing and revision of the manuscript. AF, LM, and GS contributed to the writing of clinical issue of cancer-associated cachexia and provided HNSCC patients serum. SD and FG processed the serum samples and performed qRT-PCR analysis. AS performed the bioinformatics analysis. All authors read and approved the final version of the manuscript.

## Conflict of Interest

The handling editor declared a past collaboration, though no other collaboration, with several of the authors DS, SA, and BG. The remaining authors declare that the research was conducted in the absence of any commercial or financial relationships that could be construed as a potential conflict of interest.
